# Dysphagia among geriatric trauma patients: A population-based study

**DOI:** 10.1371/journal.pone.0262623

**Published:** 2022-02-08

**Authors:** Kenny Nieto, Darwin Ang, Huazhi Liu

**Affiliations:** 1 Department of Surgery, University of Central Florida/HCA Healthcare-GME Consortium, Ocala, Florida, United States of America; 2 Department of Surgery, University of South Florida, Tampa, Florida, United States of America; 3 Department of Surgery, Division of Trauma, Ocala Regional Medical Center, Ocala, Florida, United States of America; University of Catania, ITALY

## Abstract

**Objective:**

To determine the significance of dysphagia on clinical outcomes of geriatric trauma patients.

**Methods:**

This is a retrospective population-based study of geriatric trauma patients 65 years and older utilizing the Florida Agency for Health Care Administration dataset from 2010 to 2019. Patients with pre-admission dysphagia were excluded. Multivariable regression was used to create statistical adjustments. Primary outcomes included mortality and the development of dysphagia. Secondary outcomes included length of stay and complications. Subgroup analyses included patients with dementia, patients who received transgastric feeding tubes (GFTs) or tracheostomies, and speech language therapy consultation.

**Results:**

A total of 52,946 geriatric patients developed dysphagia after admission during a 9-year period out of 1,150,438 geriatric trauma admissions. In general, patients who developed dysphagia had increased mortality, length of stay, and complications. When adjusted for traumatic brain and cervical spine injuries, the addition of mechanical ventilation decreased the mortality odds. This was also observed in the subset of patients with dysphagia who had GFTs placed. Of the three primary risk factors for dysphagia investigated, mechanical ventilation was the most strongly associated with later development of dysphagia and mortality.

**Conclusion:**

The geriatric trauma population is vulnerable to dysphagia with a large number associated with traumatic brain injury, cervical spine injury, and polytraumatic injuries that lead to mechanical ventilation. Earlier intubation/mechanical ventilation in association with GFTs was found to be associated with decreased inpatient hospital mortality. Tracheostomy placement was shown to be an independent risk factor for the development of dysphagia. The utilization of speech language therapy was found to be inconsistently utilized.

## Introduction

Geriatric trauma patients are of particular interest in trauma research due in part to the increasingly larger proportion of trauma patients they represent in the United States. What may be considered minor mechanisms of injury in younger patients may have far greater deleterious consequences to patients of advanced age who are typically frailer. In addition, many geriatric patients are exposed to polypharmacy and suffer from the cumulative effects of chronic medical conditions including neuromuscular disorders, dementia, presbycusis, and vision impairment [1, 2]. Age, traumatic brain injury (TBI), cervical spine injury (CSI), and need for mechanical ventilation have been shown to be independent risk factors for the development of dysphagia [3, 4]. Dysphagia may be cited as a complication or sequelae of trauma and non-traumatic conditions such as neurodegenerative disorders and malignancy, or in association with operative procedures (e.g., anterior approach for the repair of cervical spine injuries) [3]. The mechanisms, signs, and symptoms of dysphagia for each etiology are variable among patients.

Demonstrating the presence of dysphagia objectively is supported by screening and imaging studies. For example, the Gugging Swallowing Screen (GUSS) was utilized in a recent study on stroke patients where approximately 20.7% were diagnosed with some degree of dysphagia, and 50.9% had persistent dysfunction at time of discharge [5]. The GUSS has also been used as a predictor of aspiration [6]. Two common functional assessments of swallowing include the Fiberoptic Evaluation of Swallowing and the Video Fluoroscopic Swallowing Study (VFSS) [7]. Validation of the VFSS has been demonstrated previously, primarily in stroke and TBI literature [8]. The VFSS is useful clinically as it is scaled and can be tracked over time, and it is often considered the “gold standard” in evaluating dysphagia given its ease of reproducibility and standardization [9]. Furthermore, the VFSS can be used to identify defects in at least fifteen different components of swallowing, translating to multiple potential pathways for swallowing rehabilitation [10]. The VFSS is used extensively at the authors’ institution.

Severely ill patients as a result of traumatic and non-traumatic insults may be so physiologically impaired that prolonged mechanical ventilation or non-oral nutrition are required. Tracheostomy and transgastric feeding tubes (GFTs), such as the percutaneous endoscopic gastrostomy tube (PEG), are often utilized as a means of advancing patient care and in preparation for transition to long term care facilities.

GFT placement in the setting of TBI [11], severe dementia [12], and after trauma and other medical conditions has been reported in the literature. Regression analysis by Mandaville demonstrated that advanced age, lower Rancho Los Amigos scores, and placement of tracheostomy tube significantly increased the odds of a patient being discharged with a feeding tube [11]. Tracheostomy tubes placed for ventilatory support emergently or as a bridge from endotracheal intubation have previously been investigated in regards to the development of dysphagia [13]. Patients with tracheostomy tubes in place can still have oral nutrition and speak (e.g., with the use of a Passy Muir valve). It should be noted that aspiration can occur even with an inflated tracheostomy appliance cuff as secretions, liquids, and other particles are allowed to pass blew the vocal folds and remain trapped [14, 15].

The goal of this work is to determine the significance of dysphagia on clinical outcomes of geriatric trauma patients. We hypothesized that dysphagia would be associated with worse clinical outcomes and increased mortality. Additionally, we anticipated observing an increased rate of dysphagia with increasing age in geriatric trauma patients. We also hypothesized that GFT and tracheostomy placement would not improve outcomes, while the utilization of speech language therapist (SLP) consultation would lead to lower rates of dysphagia related complications and decreased feeding tube procedures.

## Patients and methods

### Study design and population

This is a retrospective population-based study involving trauma inpatient admissions in the State of Florida from 2010 to 2019. Publicly available and de-identified data taken from the Florida Agency for Health Care Administration (AHCA) database was used to generate results. Data for patients 65 years and older was reviewed. Trauma patients were defined according to the American College of Surgeons (ACS) National Trauma Data Bank (NTDB) standards [16]. NTDB specifies inclusion criteria based on *International Classification of Disease* (ICD), *Ninth Edition* codes 800–959.9 and *Tenth Revision* codes S00-S99, T07, T14, T20-T28, T30-T32, and T79.A1-T79.A9 [16]. Patients meeting the above criteria who had a recorded pre-admission diagnosis of dysphagia were excluded. Otherwise, all available patients were included for review. The total number of patients meeting inclusionary criteria was 1,150,438 patients. Coding used to define the phases of dysphagia include (formatted as ICD-9 / ICD-10): Oral phase (787.21 / R13.11), oropharyngeal phase (787.22 / R13.12), pharyngeal phase (787.23 / R13.13), pharyngoesophageal phase (787.24 / R13.14), and phase unspecified (787.2, 787.20, 787.29 / R13.1, R13.10, R13.19).

Specific risk factors for the development of dysphagia that will be investigated include any traumatic injury as defined by the NTDB, TBI, CSI, need for intubation or invasive mechanical ventilation, GFTs, and tracheostomy.

Traumatic brain injury is broadly defined here and is based on the Centers for Disease Control and Prevention (CDC) recommendations to best identify and include patients with TBIs from administrative data sets. These were later updated to include ICD-10 codes [17–19]. The Barell Matrix was used to select the codes we used which include ICD-9 800, 801, 803, 804, 850–854, 950.1–950.3, 959.01 [20, 21]. Codes 850–854 refer specifically to intracranial hemorrhage. In consideration of the NTDB exclusionary criteria, the ICD-10 codes that are used here include S01.0, S01.7-.9, S02.0, S02.1, S02.7-S02.9, S04, S06.0-S06.9, S07.0, S07.1, S07.8, S07.9, S09.7, and S09.9. Related injuries and mechanisms described by T01.0, T02.0, T04.0, T06.0, T90.1, T90.2, T90.4, T90.5, T90.8, T90.9 were omitted as they were included in the NTDB definition of excluded injuries [18, 21]. There were no noted limitations based on ICD-9 codes.

The definition for cervical spine injury used here is derived from ICD-9 codes that represent cervical cord (806.0–806.9 and 952.0–952.9) and cervical spine (vertebral and ligamentous) injuries (805.01-.19, 839.01-.19 and 847.0). ICD-10 code correlates include S12.0-.9, S13.1-.2, and S14.0-.1.

We defined GFTs by ICD-9 43.11 and 43.19, and ICD-10 0DH60UZ, 0DH64UZ, and 0DH67UZ, which include essentially all forms of gastrostomy tube placement including endoscopic, by laparotomy or laparoscopic technique, and fluoroscopically placed tubes. Tracheostomy tube procedural codes include ICD-9 31.1 and 31.2, and ICD-10 0B110F4, 0B113F4, and 0B114F4.

In order to include as many ventilated patients as possible in our definition of mechanical ventilation (MV), we determined that both the physical method of establishing an airway (e.g., insertion of endotracheal tube) and state of being invasively ventilated were potential methods MV could be represented clinically and by coding. We included ICD-9 96.7X (.70, .71, .72) representing the state of receiving mechanical ventilation, and 96.02 through 96.05 which represent establishment of different forms of invasive airways. ICD-10 correlates include mechanical ventilation (5A1935Z, 5A1945Z, 5A1955Z) and airways (0CHY7BZ, CHY8BZ, 0DH57BZ, 0DH58BZ, 0BH17EZ, 0BH18EZ). Given the potential for double accounting, for any one patient being considered as receiving invasive “mechanical ventilation”, appropriate Boolean operators were applied in order to negate a patient being counted a second or more times if multiple codes could identify that patient.

A literature search was performed to investigate how other authors and institutions defined dementia in their reporting. We found that there was heterogeneity in defining dementia, and documentation of codes used to compile data was often not provided [22]. Here we include the most common diagnoses and their associated ICD-9 and ICD-10 codes (formatted as ICD-9 / ICD-10) that have been used in prior research. Alzheimer disease (331.0 / G30), Lewy Body dementia and Parkinson disease with dementia (331.82 / G31.83), frontotemporal dementia (331.1 / G31.09), vascular dementia (290.40–290.43/ F01.50 and F01.51), and alcoholic and drug induced dementia (291.2 and 292.82 / F10.27, F10.97, F19.921) [23]. Senile cognitive decline or dementia (290.0 and 290.20–290.21, 797 / R41.81 and F03.90-F03.91) are also included.

### Primary outcomes

The primary outcomes in this study include the development of dysphagia and mortality.

### Secondary outcomes

Secondary outcomes of interest include length of stay (LOS), percent of patients identified as having complications, and overall complication rates. At several points additional information pertaining to long term care and nursing home discharge is demonstrated.

### Cohorts

The primary cohort reviewed was the general inclusionary population. Subset analyses were performed and include patients who developed dysphagia, those with dementia, patients who had GFT or tracheostomy procedures performed, utilization of SLP consultation, and patients who were intubated/mechanically ventilated. Our approach to reporting ventilator days was limited by the nature of ICD coding. An analysis utilizing the before and after 96 hours timeframe was made. ICD-10 codes 5A1935Z and 5A1945Z representing less than 24 hours and 24 to 96 hours, respectively, were combined to equal the ICD-9 96.71 less than 96 hours code. ICD-10 5A1955Z and ICD-9 96.72 both represent greater than 96 hours of mechanical ventilation.

## Statistical analysis

All data were analyzed using SAS version 9.4 (SAS Institute Inc., Cary, NC). Normally distributed data expressed as proportions were evaluated by χ2 tests, and continuous parametric data were compared using the t-test. Nonnormally distributed data were evaluated by Fisher’s exact test for proportions and the Wilcoxon rank-sum test for continuous data. A multivariable regression analysis was performed that adjusted for injury mechanism, International Classification Injury Severity Score (ICISS) [24], comorbidities as per the Charlson Comorbidity Index (CCI) [25], payer status, age, gender, presence of TBI, presence of cervical spine injury, and need for mechanical ventilation. The regression model also underwent reliability adjustment using a Bayesian random effects model to account for sample size variations among the different hospitals through hierarchical regression methods [26, 27].

Odds ratios were developed to characterize mortality, length of stay, percent of patients with complications, and overall complication rates. Results are demonstrated in several comparative tables. Adjustments were made in the regression models and applied to the odds ratios. These adjustments include injury mechanism, ICISS, CCI, payer, age, gender, TBI, and cervical spine injury. Mechanical ventilation was factored into the regression model as a secondary factor and reported separately.

## Results

**Fig 1:**
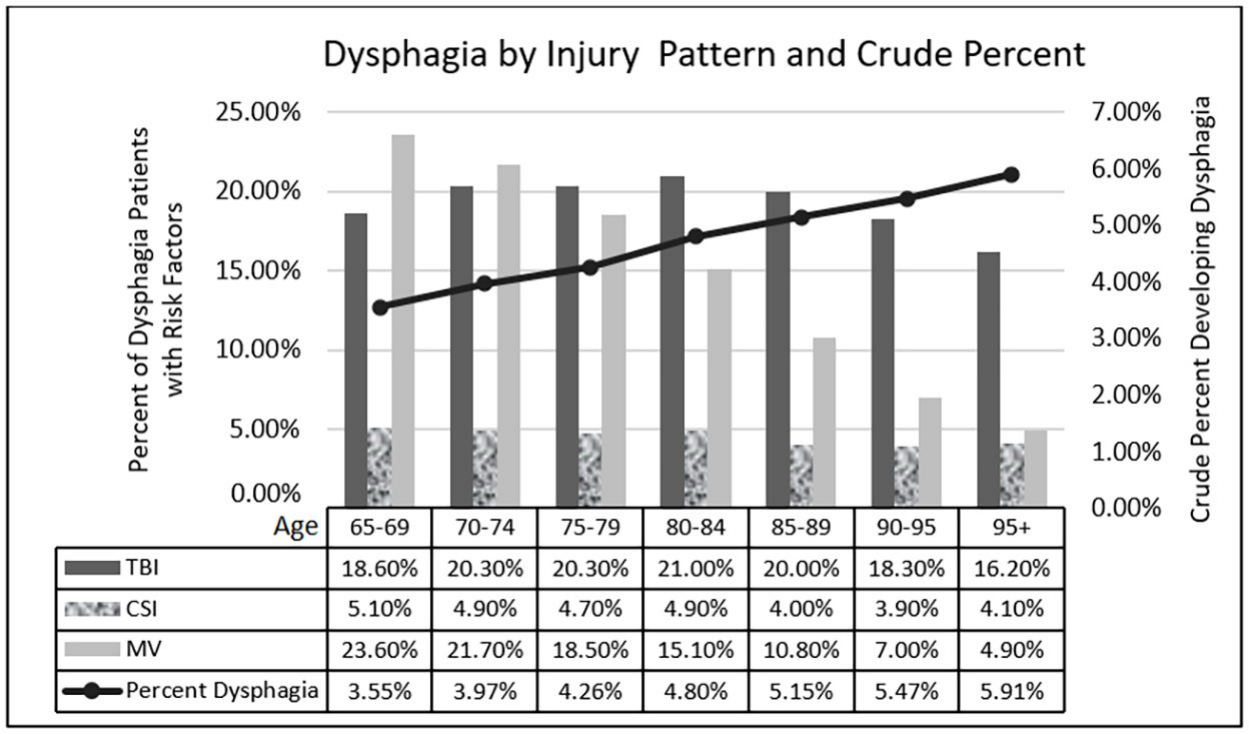
Dysphagia by injury pattern and crude percent developing dysphagia. TBI = Traumatic Brain Injury, CSI = Cervical Spine Injury, MV = Mechanical Ventilation.

Demographics are reported in Table 1. 1,150,438 patients were included after exclusionary criteria were applied. 4.6% of all these patients (n = 52,946) were found to develop dysphagia after admission. The development of dysphagia was heterogenous between age groups; as demonstrated in Fig 1, there was an increase in the crude percent of patients who developed dysphagia as age strata increased. Some form of diagnosed dementia was present in 15.4% of all patients sampled, and 23.8% of the patients who developed dysphagia had a diagnosis of dementia. The percent reporting for the dementia demographic is based on the total number of patients in the dysphagia versus without dysphagia groups. There was disproportion regarding gender of these patients as more males were found to develop dysphagia, the cause being likely multifactorial. Overall, there were more females admitted after injury, a pattern seen in reported literature [28]. P-values in Table 1 were used to determine whether those independent variables were appropriate for inclusion in our regression model, which was used in creating the remainder of our data tables. Lastly, the table demonstrates the number and percent relative to the total patients who developed dysphagia for each defined category of dysphagia by ICD classification. Although most reported cases of dysphagia were not specified by hospitals and/or the utilized databank, the most common phase of dysphagia found to be abnormal when defined was the oropharyngeal phase.

**Table 1:** Geriatric trauma patient demographics.

Demographics	Patients who Developed Dysphagia	Patients Without Dysphagia	P-value	Demographics	Patients who Developed Dysphagia	Patients Without Dysphagia	P-value
	N = 52,946	N = 1,097,492			N = 52,946	N = 1,097,492	
**Age Strata**				**Gender**			
65–69	10.5%	13.8%	<0.0001	Male	53.3%	38.3%	<0.0001
70–74	13.0%	15.2%	<0.0001	Female	46.7%	61.7%	<0.0001
75–79	15.7%	17.0%	<0.0001	**Payer**			
80–84	20.3%	19.4%	<0.0001	Medicare	91.9%	91.6%	0.01
85–89	21.5%	19.2%	<0.0001	Medicaid	0.9%	0.8%	0.02
90–94	14.1%	11.7%	<0.0001	Commercial	4.9%	5.4%	<0.0001
95+	4.9%	3.8%	<0.0001	Worker’s Compensation	0.2%	0.4%	<0.0001
**Injury Mechanism**				Government	1.2%	1.0%	<0.0001
Blunt	99.2%	99.3%	0.01	Self-Pay	1.0%	0.9%	0.11
Penetrating	0.1%	0.1%	0.17	**Dementia**			
Burn	0.7%	0.6%	0.003	Present	23.8%	15.1%	<0.0001
**Race**				Not Present	76.2%	84.9%	<0.0001
White	79.6%	81.4%	<0.0001	**Type of Dysphagia**	Number	Percent	
Black	6.6%	4.8%	<0.0001	Oral phase	1,075	2.03%	
Hispanic	11.1%	11.3%	0.07	Oropharyngeal phase	7,466	14.1%	
Asian	0.6%	0.5%	0.03	Pharyngeal Phase	971	1.83%	
Other	2.2%	2.0%	<0.0001	Pharyngoesophageal phase	150	0.28%	
				Combined (2 or more of the above listed)	302	0.57%	
				Phase Unspecified	42982	81.18%	

Fig 1 demonstrates the overall percent of patients per age strata that suffered from major risk factors for dysphagia that we investigated; TBI, CSI and MV. Mechanical ventilation was utilized less with increasing age strata. We believe this may be attributable to factors such as earlier mortality associated with fragility and the use of Do Not Resuscitate (DNR) orders, the latter of which has an established trend of increased utilization with advancing age [29].

Table 2 demonstrates the morality, length of stay (LOS), percent of patients with complications, and overall complication rates for the entire population analyzed. The data here indicates that in general there was increased mortality and worse outcomes for patients who developed dysphagia. However, a mortality benefit was demonstrated when adjustments for mechanical ventilation were made for patients who developed dysphagia.

**Table 2:** Overall mortality odds for geriatric trauma patients with dysphagia.

Geriatric Trauma Patients	With Dysphagia	Without Dysphagia	Odds Ratios	Adjusted Odds Ratios
	N = 52,946	N = 1,097,492		
**Mortality**	5.8%	2.8%	2.17 (2.09, 2.26)	1.63 (1.57, 1.70) *
				0.88 (0.84, 0.92) **
**Length of Stay (days)**	12.2 (±15.3)	5.8 (±6.7)	p<0.0001	p<0.0001
**Patients with Complication**	47.5%	26.5%	p<0.0001	p<0.0001
**Overall Complication Rates**	69.3%	33.5%	p<0.0001	p<0.0001

* Adjusted by Injury Mechanism, ICISS, CCI, payer, age, gender, TBI, cervical spine injury (CSI)

** Adjusted by Injury Mechanism, ICISS, CCI, payer, age, gender, TBI, CSI, and mechanical ventilation

Table 3 is designed analogous to Table 2, however here a subgroup analysis was performed on patients with documented dementia. Overall findings were similar regarding primary and secondary outcomes, however the mortality benefit that was seen in in the general dysphagic patients who received mechanical ventilation was not redemonstrated in the dementia cohort.

**Table 3:** Mortality odds for geriatric trauma patients with dementia who developed dysphagia.

Geriatric Trauma Patients With Dementia	Developed Dysphagia	Without Dysphagia	Odds Ratios	Adjusted Odds Ratios
	N = 12,588	N = 165,208		
**Mortality**	5.3%	2.5%	2.25 (2.07, 2.44)	1.82 (1.67, 1.99) *
				1.23 (1.12, 1.36) **
**Length of Stay (days)**	10.8 (±15.8)	5.7 (±7.4)	p<0.0001	p<0.0001
**Patients with Complication**	47.2%	31.9%	p<0.0001	p<0.0001
**Overall Complication Rates**	64.0%	37.9%	p<0.0001	p<0.0001

* Adjusted by Injury Mechanism, ICISS, CCI, payer, age, gender, TBI, cervical spine injury (CSI)

** Adjusted by Injury Mechanism, ICISS, CCI, payer, age, gender, TBI, CSI, and mechanical ventilation

An analysis of the individual risk factors (TBI, CSI, MV) and development of dysphagia in all patients included in the study is demonstrated in Table 4. There is increased dysphagia seen with all these risk factors, however it appears that after surviving a period of requiring mechanical ventilation, dysphagia was found to be significantly higher in the ventilated group. Table 5 similarly analyzes TBI, CSI, and MV however here the effect of these on mortality for the dysphagia specific cohort is investigated. It is demonstrated that for patients who developed dysphagia, overall, there was not an increase in mortality between those who did and did not have TBI. CSI patients experienced increased mortality, and similar to the overall patient population MV portended a significantly higher risk of death.

**Table 4:** Dysphagia odds among patients with major risk factors.

Presence of Risk Factor		Dysphagia Percentage	Dysphagia Odds Ratios	Dysphagia Adjusted Odds Ratios*
**Traumatic brain injury**	Yes (N = 181,417)	5.8%	1.33 (1.30, 1.36)	1.12 (1.09, 1.15)
	No (N = 969,021)	4.4%		
**Cervical spine injury**	Yes (N = 25,775)	9.3%	2.18 (2.09, 2.28)	1.86 (1.78, 1.94)
	No (N = 1,124,663)	4.5%		
**Mechanical Ventilation**	Yes (N = 56,204)	14.0%	3.77 (3.68, 3.87)	3.22 (3.13, 3.31)
	No (N = 1,094,234)	4.1%		

* Adjusted by Injury Mechanism, ICISS, CCI, payer, age, and gender

**Table 5:** Mortality odds among patients that developed dysphagia with major risk factors.

Presence of Injury Among Patients with Dysphagia		Mortality	Mortality Odds Ratios	Mortality Adjusted ORs*
**Traumatic brain injury**	Yes (N = 10,446)	5.6%	0.97 (0.88, 1.06)	0.72 (0.65, 0.80)
	No (N = 42,500)	5.8%		
**Cervical spine injury**	Yes (N = 2,398)	8.6%	1.56 (1.35, 1.81)	1.17 (1.00, 1.37)
	No (N = 50,548)	5.7%		
**Mechanic Ventilation**	Yes (N = 7,845)	17.7%	5.61 (5.20, 6.05)	5.89 (5.42, 6.40)
	No (N = 45,101)	3.7%		

* Adjusted by Injury Mechanism, ICISS, CCI, payer, age, and gender

Table 6 investigates major complications that are associated with dysphagia and the odds of patients receiving mechanical ventilation in association with them. The intention was to highlight major physiologic insults where patients may benefit from earlier intubation. The percent of patients with each condition that did or did not receive mechanical ventilation is shown. Overall, it appears the most pressing conditions leading to intubation/MV included anoxic brain injury and severe cardiac system derailments such as cardiac arrest and cardiogenic shock. However, when considering the crude percent or number of patients who were intubated given a certain risk factor, those with pulmonary conditions such as general pneumonia and aspiration pneumonia were greater.

**Table 6:** Odds ratios of Mechanical Ventilation (MV) for specific complications of patients that developed dysphagia.

Major Complications in Patients with Dysphagia	Received MV	Did Not Receive MV	Odds Ratios	Adjusted Odds Ratios*
	N = 7,845	N = 45,101		
**Neurological, Behavioral**				
**Delirium**	2.4%	2.2%	1.11 (0.95, 1.30)	1.05 (0.89, 1.25)
**Stroke**	6.3%	5.5%	1.15 (1.04, 1.27)	1.22 (1.09, 1.36)
**Anoxic brain injury**	6.7%	0.4%	16.67 (14.10, 19.71)	13.94 (11.65, 16.69)
**Cardiovascular**				
**Acute Myocardial Infarction**	3.7%	1.6%	2.44 (2.13, 2.81)	2.32 (1.98, 2.70)
**Cardiac arrest**	11.4%	0.5%	26.78 (23.03, 31.15)	18.13 (15.40, 21.34)
**Atrial Fibrillation**	31.8%	25.6%	1.35 (1.29, 1.43)	1.48 (1.40, 1.57)
**Congestive Heart Failure**	26.2%	16.6%	1.78 (1.68, 1.88)	1.95 (1.83, 2.08)
**Pulmonary**				
**Pneumonia**	40.0%	13.6%	4.24 (4.02, 4.46)	3.79 (3.58, 4.01)
**Aspiration Pneumonia**	34.8%	16.0%	2.81 (2.66, 2.96)	2.84 (2.68, 3.01)
**Empyema (Pyothorax)**	0.8%	0.2%	4.01 (2.89, 5.56)	3.01 (2.09, 4.34)
**Iatrogenic Pneumothorax**	1.2%	0.2%	6.52 (4.83, 8.78)	5.36 (3.86, 7.46)
**ARDS**	13.2%	1.6%	9.32 (8.45, 10.29)	8.04 (7.21, 8.96)
**Hematologic and Immune**				
**Pulmonary embolism**	2.7%	1.1%	2.57 (2.18, 3.02)	2.34 (1.95, 2.80)
**DVT (any extremity)**	5.7%	2.4%	2.48 (2.22, 2.78)	2.20 (1.94, 2.49)
**Gastrointestinal**				
**Gastrointestinal bleeding**	3.4%	1.5%	2.30 (1.99, 2.66)	2.20 (1.88, 2.58)
**Clostridium Difficile Infection**	4.2%	1.9%	2.26 (1.99, 2.57)	2.06 (1.78, 2.37)
**Peritonitis**	0.4%	0.1%	5.96 (3.61, 9.85)	4.46 (2.56, 7.76)
**Abdominal Compartment Syndrome**	0.1%	0.004%	31.66 (7.02, 142.87)	6.68 (1.27, 35.02)
**Musculoskeletal (& Connective Tissue)**				
**Extremity Compartment Syndrome**	0.2%	0.04%	4.32 (2.21, 8.44)	2.44 (1.15, 5.17)
**Rhabdomyolysis**	3.3%	2.5%	1.37 (1.20, 1.57)	1.40 (1.21, 1.63)
**Genitourinary**				
**Urinary Tract Infection**	26.4%	25.3%	1.06 (1.00, 1.12)	1.20 (1.13, 1.27)
**Acute Kidney Failure**	29.7%	19.0%	1.80 (1.71, 1.90)	1.79 (1.69, 1.91)
**Integumentary Associated**				
**Fasciitis (unspecified location)**	0.05%	0.02%	2.88 (0.87, 9.55)	2.40 (0.63, 9.20)
**Decubitus Ulcer (≥Stage 2)**	12.2%	8.1%	1.59 (1.48, 1.72)	1.55 (1.42, 1.68)
**Endocrine, Nutritional, and Metabolic Disease**				
**Diabetes with Ketoacidosis or Hyperosmolar Coma**	0.4%	0.1%	2.60 (1.66, 4.07)	2.64 (1.64, 4.25)
**Moderate and Severe Protein-Calorie Malnutrition**	12.7%	8.7%	1.54 (1.43, 1.65)	1.38 (1.27, 1.50)
**Miscellaneous Infectious/Critical Care**				
**SIRS with Organ Dysfunction (Infection and Non-infectious)**	0.8%	0.3%	2.78 (2.06, 3.76)	1.84 (1.31, 2.57)
**Septic Shock**	13.3%	1.5%	10.09 (9.13, 11.16)	8.59 (7.69, 9.59)
**Cardiogenic Shock**	3.0%	0.2%	14.79 (11.63, 18.80)	14.89 (11.49, 19.31)
**Hemorrhagic Shock**	3.4%	0.6%	6.32 (5.31, 7.53)	3.88 (3.18, 4.72)
**Infection After a Procedure**	0.3%	0.2%	1.22 (0.76, 1.96)	0.69 (0.41, 1.18)

* Adjusted by Injury Mechanism, ICISS, CCI, payer, age, and gender, mortality

ARDS is Acute Respiratory Distress Syndrome, DVT is Deep Vein Thrombosis

GFTs, tracheostomies, and SLP therapist involvement on patient outcomes is next investigated. Table 7 demonstrates the total number and percent of patients with and without dysphagia who had tracheostomies and feeding tubes placed. Both interventions were found to be more frequently utilized in patients who developed dysphagia.

**Table 7:** Frequency of tracheostomy and transgastric feeding tube placement.

Procedure	Patients With Dysphagia	Patients Without Dysphagia	P-Values
	N = 52,946	N = 1,097,492	
Tracheostomy	3.0%	0.5%	P<0.001
Gastric Feeding Tube	16.2%	0.6%	P<0.001

Table 8 investigates associations between patients who developed dysphagia and had GFTs placed. The data suggest there is an increased risk of mortality for patients in general, however, when adjustments are made for MV there was a mortality benefit observed. Otherwise, feeding tubes are associated with longer LOS and more complications. GFTs appear to be a risk factor for discharging to a nursing home or long-term care facility. Table 9 demonstrates that tracheostomies and/or conditions leading to tracheostomy being required are significant risk factors for the development of dysphagia, mortality, LOS, and complications.

**Table 8:** Outcomes in patients with dysphagia who received transgastric feeding tubes (GFTs).

Geriatric Trauma Patients Who Developed Dysphagia	With GFTs	Without GFTs	Odds Ratios	Adjusted Odds Ratios
	N = 8,588	N = 44,358		
**Mortality**	6.9%	5.6%	1.25 (1.14, 1.37)	1.09 (0.99, 1.20) *
				0.71 (0.64, 0.78) **
**Length of Stay (days)**	19.6 (±26.5)	10.7(±11.5)	p<0.0001	p<0.0001
**Patients with Complication**	61.5%	44.8%	p<0.0001	p<0.0001
**Overall Complication Rates**	104%	62.6%	p<0.0001	p<0.0001
**Patients Discharging to Nursing & Long-Term Care Facilities**	59.6%	43.6%	1.91 (1.81, 2.02)	2.00 (1.90, 2.12) *
				2.03 (1.92, 2.15) **

* Adjusted by Injury Mechanism, ICISS, CCI, payer, age, gender, TBI, cervical spine injury (CSI)

** Adjusted by Injury Mechanism, ICISS, CCI, payer, age, gender, TBI, CSI, and mechanical ventilation

**Table 9:** Outcomes of geriatric trauma patients who had tracheostomy placed.

Geriatric Trauma Patients	Tracheostomy Performed	No Tracheostomy	Odds Ratios	Adjusted Odds Ratios
	N = 6,884	N = 1,143,554		
**Developed Dysphagia**	22.8%	4.5%	6.26 (5.91, 6.63)	4.76 (4.48, 5.06) *
**Mortality**	13.6%	2.8%	5.40 (5.04, 5.79)	1.33 (1.22, 1.44) *
**Length of Stay (days)**	30.4 (±28.0)	5.9 (±6.9)	p<0.0001	p<0.0001
**Patients with Complication**	91.4%	27.1%	p<0.0001	p<0.0001
**Overall Complication Rates**	205%	34.2%	p<0.0001	p<0.0001

* Adjusted by Injury Mechanism, ICISS, CCI, payer, age, gender, TBI, cervical spine injury

SLP therapist involvement with dysphagic patients is investigated in Table 10. Notable findings include increased feeding tube and decreased tracheostomy utilization, both of which were significant. There were patients identified with aspiration pneumonia who did not have SLP consultation. Regarding duration of mechanical ventilation, for those patients who developed dysphagia it was found that in those with shorter times on the ventilator (<96 hours, adjusted OR 1.58), SLP was utilized more frequently than not, and less for those with longer times on support (>96 hours, adjusted OR 0.75). LOS was significantly longer and there were more complications and long-term care facility discharges associated with patients receiving SLP evaluation and treatment.

**Table 10:** Associations of Speech Language Therapist (SLP) utilization on outcomes of geriatric trauma patients who developed dysphagia after traumatic injury.

Geriatric Trauma Patients with New Onset Dysphagia	With SLP consultation	Without SLP consultation	Odds Ratios	Adjusted Odds Ratios
	N = 38,212	N = 14,734		
**Gastric feeding tube placed**	18.4%	10.7%	1.88 (1.77, 1.99)	1.82 (1.72, 1.93) *
				1.87 (1.77, 1.98) **
**Tracheostomy placed**	2.3%	4.6%	0.50 (0.45, 0.56)	0.45 (0.40, 0.50) *
				0.39 (0.35, 0.44) **
**Aspiration Pneumonia**	20.6%	14.0%	1.60 (1.52, 1.69)	1.60 (1.52, 1.69) *
				1.62 (1.53, 1.71) **
**Ventilation <96 hours**	7.9%	5.2%	1.56 (1.44, 1.70)	1.58 (1.45, 1.72) *
**Ventilation > 96 hours**	7.2%	9.1%	0.77 (0.72, 0.82)	0.75 (0.69, 0.80) *
**Length of Stay (days)**	13.3 (±15.6)	9.2 (±14.2)	P<0.0001	p<0.0001
**Patients with Complications**	49.1%	43.5%	P<0.0001	p<0.0001
**Overall Complication Rate**	70.9%	65.0%	P<0.0001	p<0.0001
**Patients Discharged to Nursing and Long-Term Care Facilities**	47.5%	40.5%	1.33 (1.28, 1.38)	1.35 (1.29, 1.40) *
				1.35 (1.29, 1.40) **

* Adjusted by Injury Mechanism, ICISS, CCI, payer, age, gender, TBI, cervical spine injury (CSI)

** Adjusted by Injury Mechanism, ICISS, CCI, payer, age, gender, TBI, CSI, and mechanical ventilation

The overall effects of duration of mechanical ventilation on mortality, LOS, and complications are demonstrated in Table 11. LOS and complications were increased, consistent with prior literature [7]. Conditions leading to shorter mechanical ventilation utilization were associated with increased mortality.

**Table 11:** Mortality, development of dysphagia, and complications of geriatric trauma patients who were mechanically ventilated.

Geriatric Trauma Patients	Mechanical Ventilation Less than 96 Hours	Mechanical Ventilation Greater than 96 Hours	Odds Ratios	Adjusted Odds Ratios
**Mortality**	35.8%	28.1%	0.70 (0.68, 0.73)	0.70 (0.67, 0.72) *
				0.70 (0.67, 0.73) **
**Length of Stay (days)**	9.0 (±9.2)	23.7 (±27.8)	P<0.0001	p<0.0001
**Developed Dysphagia**	10.8%	19.0%	1.93 (1.84, 2.03)	2.02 (1.92, 2.13) *
				2.02 (1.92, 2.13) **
**Overall Complication Rates**	157%	202%	P<0.0001	p<0.0001

* Adjusted by Injury Mechanism, ICISS, CCI, payer, age, gender, traumatic brain injury

** Adjusted by Injury Mechanism, ICISS, CCI, payer, age, gender, cervical spine injury

## Discussion

This study stemmed from the interest of the trauma program at the authoring institution to investigate the implications dysphagia has on geriatric trauma patients. Our institution is located central to several large retirement communities in the state of Florida, and the average age of patients admitted to our trauma service is 60 years old. Examples of pre-hospital living situations for these patients include independent residence, living with family, homeless, skilled nursing and long-term care facilities, and rehabilitation centers. Our institution has staffed SLP therapists who are available daily, and they are also active in a multidisciplinary “tracheostomy team.” Rancho Los Amigos assessment, swallowing rehabilitation, and VFSS are a few of the services they provide trauma and non-trauma patients. Practice management guidelines have been established to help ensure patients at risk for dysphagia are evaluated. The development of dysphagia has a significant impact on patient and family quality of life, as these patients are more likely to be institutionalized, need more family care, and suffer from complications such as pneumonia [5].

There were two major unexpected findings in relation to the primary outcomes in this study. First was the finding of decreased mortality in the general geriatric patient who developed dysphagia after adjustments were made to include TBI, CSI, and mechanical ventilation (Table 2). Second was decreased mortality in dysphagic patients who had feeding tubes placed, after adjustments were made for mechanical ventilation. Explanations for these findings revolve around considering some protective factor intubation/mechanical ventilation has these patients who would have otherwise been expected to have higher mortality. Another explanation is that these patients had a higher rate of discharge to secondary care facilities, as the placement of patients on ventilators may qualify them for earlier disposition to long term acute care facilities and skilled nursing facilities that have ventilator management capabilities.

Regarding secondary outcomes, the development of dysphagia was associated with significantly longer length of stay and complications in all subgroup analyses performed. When considering TBI, CSI, and need for mechanical ventilation as risk factors, all were associated with an increased risk of developing dysphagia. Additively, these three risk factors accounted for 70.9% of the patients who were found to develop dysphagia overall. The need for mechanical ventilation portended a significantly higher risk. We would like to present that the data for Table 4 was compiled twice. In the first iteration MV was defined only by endotracheal intubation. The second iteration published here was by our expanded definition for MV. The percent with MV who developed dysphagia was 10% vs. 14% in our final definition, and the adjusted odds ratio was 2.76 vs. 3.22. A broader definition effectively captured more patients.

A dilemma that often arises in disposition planning is determining an appropriate mode of alimentation for patients who are significantly dysphagic and dependent on non-oral means of nutrition. In fact, as highlighted in prior publications, facilities often require durable feeding access (e.g., gastrostomy tube) as opposed to allowing continuation of nasoenteric feeding. Pleasure feeding is a palliative option [30]. It has been demonstrated that there is no survival advantage of having GFTs placed. Periprocedural complications are common, the most common and feared being aspiration pneumonia [31–34]. Others include trans-colonic tube placement and tube dislodgement [34]. Here we do not differentiate between techniques used for gastrostomy tube placement due to limitations in ICD coding, however later research could use data sets that utilize CPT codes which would allow for statistical comparison between techniques. Contrary to our hypothesis that SLP therapy would be associated with reduced feeding tube utilization, increased feeding tube procedures were noted. The clinical context of this hypothesis is a trend in the literature calling for the reconsideration of meaningful outcomes and complications associated with procedures performed on at risk geriatric patients when there are noninvasive options [35]. PEG tubes are often being used as part of a bridge between acute-care and long-term care facilities, and questions have arisen in regards to the risk-benefit-ratio of these procedures and associated complications [36, 37]. There is no high quality evidence to suggest that PEG tube placement imparts any long term-survival benefit, and more frequently clinicians are asked to re-evaluate the ethical principles and indications guiding a decision for invasive feeding access [12]. Potential complications associated with GFTs are many and occur frequently, including immediate and delayed procedural complications, wound complications, aspiration, and tube malfunction [38]. Aspiration pneumonia is the most common cause of death after PEG tube placement, patients with a prior history of aspiration pneumonia being at highest risk [33]. The fundamental principle is that feeding tubes do not modify the processes causing dysphagia. In addition, our data demonstrates a non-negligible number of geriatric trauma patients with dysphagia who did not have SLP involvement even with documented aspiration pneumonia. This could serve as an additional area for hospital quality improvement.

The current standard approach to the management of a patient with a tracheostomy includes a multidisciplinary team consisting of a SLP therapist, nurses, respiratory therapists, and physicians that monitor patients and provide goal directed care. Implementing such teams has been shown to not only reduce hospital length of stay, but also decrease intensive care unit days and total time a tracheostomy is used [39]. Improvement in swallowing function can be achieved in appropriately selected patients [40]. It should be noted that decannulation does not necessarily lead to improved swallowing function [41]. The association between dysphagia and tracheostomy is complex and will not be further elaborated. An important extrapolation includes the association between frequency of the development of dysphagia and longer length of intubation (greater than 24–48 hours); this effect is more pronounced with increasing age [42].

There is prior literature that demonstrates longer periods of intubation/mechanical ventilation are associated with a higher risk of developing dysphagia. Our study supports these findings (Table 11). It should be noted to caution interpretation and consider outcomes observed here are likely related to sequelae of trauma rather than due to mechanical ventilation itself. At first glance the odds ratio for mortality appears to favor a longer time on a ventilator. However, interpretation should consider the reality that patients with more severe traumatic injuries are more likely to have early mortality and perhaps not even survive the first 24–48 hours of admission. The value demonstrated for increased mortality with ventilation <96 hours is likely a factor of secondary and tertiary deaths within the early timeframe of hospitalization [43, 44]. This table overall serves to highlight the severity of illness secondary to injury in geriatric patients, and sequela conditions requiring ventilation have on outcomes.

Several important limitations were identified when designing this study, and others that became apparent on review of the results. When drawing conclusions in reference to the risk factors for dysphagia, it needs to be cautioned that temporal relationships cannot be made, and an even more egregious error would be to assume causality. Perhaps the two most important examples are those that were contrary to our pre-investigation hypotheses; the MV adjusted mortality for the general studied patient who developed dysphagia in Table 2, and the MV adjusted mortality associated with GFTs in Table 8. Unexplored factors that are apparently protective towards decreasing inpatient mortality are demonstrated at the population-based level. The role mechanical ventilation plays in these findings may be related to disease modification such as airway protection from aspiration, allowing for correction of physiologic disturbances such as acidosis or hypoxia, or even playing a role in earlier discharge to secondary care centers. In this regard, perhaps earlier intubation should be considered when the first signs of major systematic complications are encountered, rather than awaiting laboratory and imaging studies that prove a patient is deteriorating. It cannot be determined from the administrative data set used whether invasive airways were established immediately on arrival to hospitals, or after complications developed.

Data is limited fundamentally by the way each patient experience is coded. Our method to reduce omission of any significant number of patients was to carefully construct each definition utilized. The CDC reports the same difficulty in creating their definition of TBI to the extent that numerous validation studies were funded to ensure research best reflects clinical reality [21]. There is also literature investigating the representation of dysphagia coding in databases, which is noted to have high false negative rates which suggests undercoding [45]. Table 11 would ideally have been expanded to include more time intervals in an attempt to establish a Kaplan Meier curve demonstrating the relationship between ventilator time on mortality and dysphagia. This was limited by the use of a generalized administrative dataset but would be a useful study to elaborate established literature. Finally, knowledge of DNR or even Do Not Intubate status would have been useful in our analysis but was not available in the Florida AHCA dataset.

Unique features of our study include it being a population-based analysis of dysphagia after trauma, in addition to reporting specifically on geriatric patients. Relationships between TBI, CSI, MV, and time of mechanical ventilation with the development of dysphagia were also investigated at a population level. Several of our hypothesis were disproven, notably mortality associated with GFT placement and GFT utilization in association with SLP consultation. The demonstrated mortality benefit when adjustments were made for mechanical ventilation was not expected.

## Conclusion

Our study demonstrates that geriatric trauma patients are at risk for developing dysphagia after various traumatic injuries, intubation, and in association with feeding tubes and tracheostomy placement. Furthermore, the risk of developing dysphagia increases with age along the spectrum of elderly patients. Overall, dysphagia among geriatric trauma patients is associated with increased mortality. However, except for patients with dementia, intubation/mechanical ventilation appears to have a protective role and earlier intubation should be considered at the earlier stages of physiologic decompensation. Patients who developed dysphagia after TBI did not have significantly higher mortality than those who did not. However, cervical spine injury is a significant risk factor for both dysphagia and mortality. The disease processes and trauma leading to requirement of intubation/mechanical ventilation are believed to be major contributors to MV being observed as an independent risk factor for the development of dysphagia and mortality.

Finally, while GFTs and tracheostomies are generally associated with poor outcomes, a protective factor was observed when mechanical ventilation was accounted for among patients with GFTs. GFT placement continues to be a controversial part of patient disposition in our communities; however, it may have a beneficial role in patients who are already mechanically ventilated. Future studies specific to geriatric trauma can elaborate on existing knowledge of the relationship between time interval of mechanical ventilation and rate of dysphagia, relationship of DNR status and mortality, and develop protocols that provide patients with increased access to SLP for prevention and management of aspiration.
